# *Faecalibacterium prausnitzii* A2-165 metabolizes host- and media-derived chemicals and induces transcriptional changes in colonic epithelium in GuMI human gut microphysiological system

**DOI:** 10.20517/mrr.2024.14

**Published:** 2024-05-22

**Authors:** Yu-Ja Huang, Caroline A. Lewis, Charles Wright, Kirsten Schneider, John Kemmitt, David L. Trumper, David T. Breault, Omer Yilmaz, Linda G. Griffith, Jianbo Zhang

**Affiliations:** ^1^Department of Biological Engineering, Massachusetts Institute of Technology, Cambridge, MA 02139, USA.; ^2^Whitehead Institute for Biomedical Research, Cambridge, MA 02142, USA.; ^3^current address: UMass Chan Medical School, Program in Molecular Medicine, Worcester, MA 01605, USA.; ^4^Department of Mechanical Engineering, Massachusetts Institute of Technology, Cambridge, MA 02139, USA.; ^5^Department of Pediatrics, Harvard Medical School, Boston, MA 02115, USA.; ^6^Center for Gynepathology Research, Massachusetts Institute of Technology, Cambridge, MA 02139, USA.; ^7^Swammerdam Institute for Life Sciences, University of Amsterdam, Amsterdam 1098 XH, the Netherlands.; ^8^Tytgat Institute for Liver and Intestinal Research, Amsterdam Gastroenterology, Endocrinology and Metabolism, Amsterdam UMC, Location Academic Medical Center, Amsterdam 1105 BK, the Netherlands.

**Keywords:** *Faecalibacterium prausnitzii* A2-165, colonic epithelium, metabolomics, microphysiological system, host-microbe interaction

## Abstract

**Aim:** Recently, a GuMI gut microphysiological system has been established to coculture oxygen-intolerant *Faecalibacterium prausnitzii* (*F. prausnitzii*) A2-165 with organoids-derived primary human colonic epithelium. This study aims to test if this GuMI system applies to different donors with different healthy states and uses metabolomics to reveal the role of gut microbes in modulating host- and diet-derived molecules in the gut lumen.

**Methods:** Organoids-derived colonic monolayers were generated from an uninflamed region of diverticulitis, ulcerative colitis, and Crohn’s disease patients and then integrated into the GuMI system to coculture with *F. prausnitzii* A2-165 for 2 to 4 days. Apical media was collected for metabolomic analysis. Targeted metabolomics was performed to profile 169 polar chemicals under three conditions: conventional static culture without bacteria, GuMI without bacteria, and GuMI with *F. prausnitzii*. The barrier function of monolayers was measured using transepithelial resistance.

**Results:** GuMI successfully cocultured patient-derived monolayers and *F. prausnitzii* for up to 4 days, with active bacterial growth. Introducing flow and oxygen gradient significantly increases the barrier function, while exposure to *F. prausnitzii* slightly increases the barrier function. Targeted metabolomics screened 169 compounds and detected 76 metabolites, of which 70 significantly differed between at least two conditions. *F. prausnitzii* significantly modulates the levels of nucleosides, nucleobases, and amino acids on the apical side. Further analysis suggests that *F. prausnitzii* changes the mRNA level of 260 transcription factor genes in colonic epithelial cells.

**Conclusion:** The GuMI physiomimetic system can maintain the coculture of *F. prausnitzii* and colonic epithelium from different donors. Together with metabolomics, we identified the modulation of *F. prausnitzii* in extracellular chemicals and colonic epithelial cell transcription in coculture with human colonic epithelium, which may reflect its function in gut lumen *in vivo*.

## INTRODUCTION

Microbial activities influence the host by modulating chemical structures and producing bioactive compounds. Gut microbes ferment complex molecules such as polysaccharides to short-chain fatty acids. Additionally, gut microbes convert compounds derived from the host and diet to their metabolites. For example, primary bile acids, synthesized in the liver and released to the gut, are converted to secondary bile acids by the gut bacteria and reabsorbed in the colon^[1]^. Dietary amino acids such as tryptophan can be metabolized by gut microbes and circulated in the bloodstream^[2,3]^. These metabolites can target G-protein-coupled receptors and trigger inflammation. Gut microbes can also convert dietary toxicants to metabolites with lower cytotoxicity and mutagenicity^[4,5]^.

*Faecalibacterium* is a genus of strictly anerobic, extremely oxygen-sensitive (EOS), Gram-positive, rod-shaped, nonmotile, and non-spore-forming bacteria^[6]^*.* It is highly abundant and prevalent in adult human gut microbiota, accounting for around 5% of the human gut microbiota^[7]^, and 85% of the gut samples^[8]^. The species *Faecalibacterium prausnitzii* (*F. prausnitzii*) is strongly and reversely associated with inflammatory bowel diseases^[9]^. Mechanistically, *F. prausnitzii* was found to have anti-inflammatory effects through several modes of action^[10-12]^. Butyrate production is a hallmark of *F. prausnitzii*. Butyrate’s effects on modulating host gene expression and inflammation have been extensively studied using cell lines, organoids, animal models, and clinical trials^[11]^. More recently, metabolomics has unveiled additional novel metabolic signatures, such as 5-aminosalicylic acid, which contribute to the anti-inflammatory effects of *F. prausnitzii*^[13]^, suggesting that metabolomics could be an effective approach to identifying new metabolites^[13]^. *F. prausnitzii* was found to release amino acids in a fully defined medium^[14]^. These studies show that *F. prausnitzii* possesses many previously unknown metabolic functions by producing bioactive metabolites.

Recently, gut microphysiological systems have been developed to coculture human and strictly anerobic bacterial species under physiologically relevant oxygen gradient and flow^[15-18]^. These systems offer a new tool to study the causal link between microbes and their effects on the host. GuMI has recently been shown to culture EOS gut microbe *F. prausnitzii* with primary human colon epithelium of one donor^[15]^. *F. prausnitzii* actively produces butyrate and exerts anti-inflammatory effects by modulating the expression of inflammation-related genes in the NF-κB pathway^[15]^. However, it is unclear whether the GuMI system is feasible for other donors and what metabolites beyond butyrate are modulated by *F. prausnitzii*. Here, we tested the coculturing of *F. prausnitzii* and colonic epithelium from multiple donors. We further applied targeted metabolomics to identify metabolites produced by *F. prausnitzii* in the GuMI system and compared the metabolite profiles with that in GuMI without bacteria and under conventional Static culture.

## METHODS

### Bacterial culture

*F. prausnitzii* A2-165 (DSM 17677 or JCM 31915, now renamed *F. duncaniae* A2-165^[19]^) was obtained from the Harvard Digestive Disease Center. Bacterial culturing and identity confirmation were performed according to a previous study^[5]^. Bacteria from glycerol stock were plated in yeast casitone fatty acid (YCFA) agar (Anaerobe Systems, AS-675). After 24-48 h being cultured at 37 °C in the incubator inside the anerobic chamber (Coy Laboratory), a colony was picked and cultured in liquid YCFA medium (Anaerobe Systems, AS-680). O_2_ in the anerobic chamber was constantly removed by the Palladium Catalyst (Coy Laboratory, #6501050), which was renewed biweekly by incubating in the 90 °C oven for two days.

### Organoids, monolayers, and GuMI experiments

The organoid was derived from patients following the protocol approved by the Institutional Review Board of Boston Children’s Hospital (protocol number IRB-P00000529), the Koch Institute Institutional Review Board Committee, and the Massachusetts Institute of Technology Committee, and informed consent was obtained from the patients. The monolayers were prepared according to the protocols described previously^[16,20]^ with more details in the Supplementary Material.

### TEER measurement

EndOhm-12 chamber with an EVOM2 meter (World Precision Instruments) was used to measure the TEER values.

### Sample preparation and targeted metabolomic analysis

Media collected from the apical compartment of GuMI was centrifuged to pellet the bacterial cells. Five microliters of the supernatant were mixed with 195 μL extraction mix (50:30:20 methanol:acetonitrile:water containing 0.2 μg/mL internal standards (Metabolomics Amino Acid Mix MSK-A2-1.2, Cambridge Isotope Laboratories, Inc.) obtained from the core facility and stored at -20 °C), followed by media extraction protocol for polar metabolites (50:30:20 method). Briefly, the mixture was vortexed for 5 min in the cold room and then centrifuged for 10 min at maximal speed and 4 °C. After that, supernatants were transferred to a new 1.5-mL tube and stored at -80 °C until analysis.

For liquid chromatography-mass spectrometry (LC-MS) analysis, 2 μL of each sample was injected onto a ZIC-pHILIC 2.1 mm × 150 mm (5 μm particle size) column (Millipore Sigma), connected to a Q-Exactive orbitrap mass spectrometer. The mobile phases were 20 mM ammonium carbonate, 0.1% ammonium hydroxide (buffer A), and pure acetonitrile (buffer B). The chromatographic gradient was run at a flow rate of 0.150 mL/min as follows: 0-20 min: linear gradient from 80% to 20% B, 20-20.5 min: linear gradient from 20% to 80% B; 20.5-28 min: hold at 80% B. The mass spectra were recorded in full-scan and polarity-switching mode with the following parameters: spray voltage at 3.0 kV, the heated capillary at 275 °C, and the HESI probe at 350 °C. The sheath gas flow was set to 40 units, the auxiliary gas flow was set to 15 units, and the sweep gas flow was set to 1 unit. The MS data acquisition was performed in a range of 70-1,000 m/z, with the resolution set at 70,000, the automatic gain control target at 106, and the maximum injection time at 80 msec. Relative quantitation of polar metabolites was performed with Tracefinder 4.1 (Thermo Fisher Scientific) by extracting ion chromatograms using a 5 ppm mass tolerance and referencing an in-house retention-time library of chemical standards. Peak area ratios for the analytes were calculated, in which the raw peak area for each metabolite was divided by the raw peak area of the appropriate internal standard, i.e., the isotopically labeled amino acids [Supplementary Tables 1 and 2].

### Metabolomic data analysis

The targeted metabolomic data were analyzed using Metaboanalyst (https://www.metaboanalyst.ca/) following the protocol^[21]^. Briefly, the data (peak to internal standard ratio, Supplementary Tables 1 and 2) were uploaded, normalized by sum, transformed (log transformation, base 10), and auto-scaled (mean-centered and divided by the standard deviation of each variable). After that, the normalized data were clustered using the following parameters (distance calculation, Euclidean; clustering method, Ward; color contrast, default) and visualized as clustered heatmap.

### Transcription factor analysis

A list of transcription factors was retrieved from multiple databases^[22,23]^ and compiled in Supplementary Tables 3 and 4. The gene expression of these transcription factors in colonic epithelial cells was retrieved from our previous study^[15]^ and compared under different conditions [Supplementary Tables 3 and 4]. Briefly, a comparison was performed between GuMI with no bacteria (GuMI-NB) and Static culture and between GuMI with *F. prausnitzii* (GuMI-FP) and GuMI-NB. Fold change and adjusted p values were calculated as previously reported^[15]^ and used to identify the effects of microenvironment and bacterium *F. prausnitzii*. A gene is deemed significantly changed in one comparison if its adjusted *P*-value is less than 0.05 and the absolute value of fold change exceeds 2.

## RESULTS

### GuMI is versatile for coculture of bacteria and colonic epithelium derived from multiple donors

**Figure 1:**
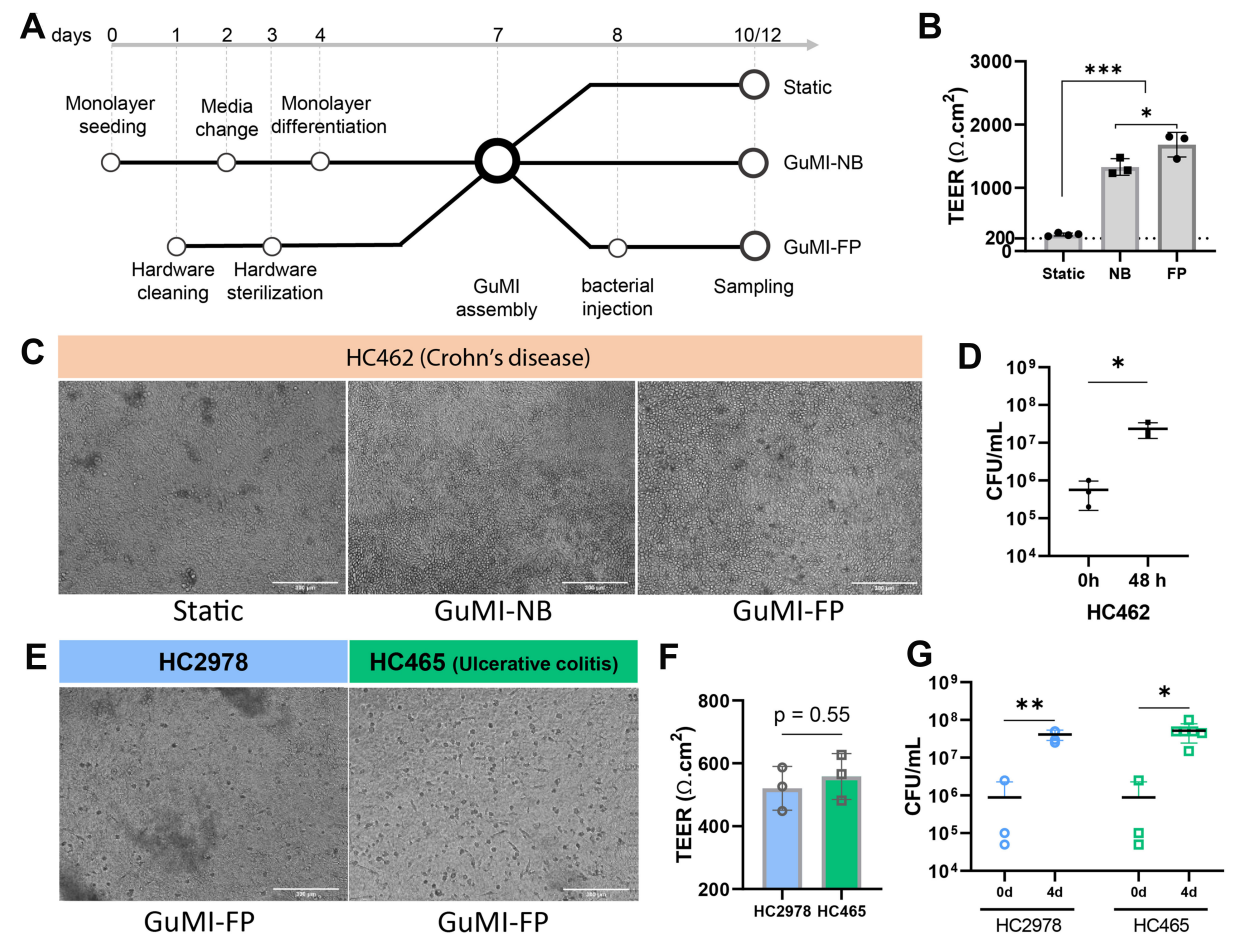
GuMI can coculture *F. prausnitzii* A2-165 with colonic epithelium from multiple donors for up to 4 days. (A) The general workflow of the GuMI experiment includes monolayer seeding, hardware preparation, GuMI assembly, bacterial injection, and sampling; (B) TEER value of monolayers at 48 hours after bacterial injection under static condition (Static), GuMI-FP or GuMI-NB. The monolayers were derived from an uninflamed/unaffected region in the transverse colon from donor H462, a 15-year-old male with CD. *P < 0.05, ***P < 0.001, one-way ANOVA was performed with Tukey’s multiple comparisons test; (C) Brightfield images of monolayers at the end of the GuMI experiment in Static, GuMI-NB, and GuMI-FP; (D) Growth of *F. prausnitzii* after 48 h of coculture in GuMI. *P < 0.05 two-tailed unpaired *t*-test; (E) Brightfield images of monolayers. Coculture of *F. prausnitzii* with two donors, HC2978 and HC465 (ulcerative colitis patient), was maintained for four days. Dendritic cells and macrophages were included underneath the monolayers; (F) TEER values of monolayers in GuMI. *P* = 0.55, two-tailed unpaired *t*-test; (G) Bacterial density in the apical compartment of GuMI after four days of coculture compared to the inoculum (0d). Note that the same inoculum (0d) applies to HC2978 and HC465. *P < 0.05, **P < 0.01, two-tailed unpaired *t*-test. TEER: Transepithelial electric resistance; GuMI-FP: GuMI with *F. prausnitzii*; GuMI-NB: GuMI without bacteria.

We previously demonstrated a successful coculture of *F. prausnitzii* with human colonic epithelial cells^[15]^ and innate immune cells^[16]^ under physiologically relevant oxygen gradient and flow. The colonic epithelium was derived from healthy tissue. To test whether GuMI can accommodate bacteria-host coculture from multiple donors, we prepared organoid-derived monolayers from the uninflamed colonic region of the patient with Crohn’s disease based on the previously reported protocol^[20]^. Monolayers were cultured under three conditions for 4 days [Figure 1A], i.e., Static culture, GuMI-NB, and GuMI-FP. Interestingly, the TEER values of monolayers in GuMI were significantly higher than those in Static culture (*P* < 0.001, Figure 1B). Additionally, TEER values in GuMI-FP were higher than those in GuMI-NB (*P* < 0.05, Figure 1B). In agreement, we observed dark spots in the monolayers under Static culture, whereas there were fewer in the monolayers in GuMI-NB and GuMI-FP [Figure 1C]. Correspondingly, *F. prausnitzii* actively grew in GuMI, with live bacterial cell density increasing from 5.7 × 10^5^ to 2.3 × 10^7^ CFU/mL at day 2 after bacterial injection [Figure 1D]. For a donor of ulcerative colitis (HC465, Figure 1E), we compared head-to-head with a non-UC donor HC2978 in coculturing *F. prausnitzii* for four days in GuMI with dendritic cells and macrophages^[16]^. TEER values are not significantly different between the two donors [Figure 1F], and *F. prausnitzii* grew significantly after four days of coculture [Figure 1G]. Although these pilot data suggest that GuMI can accommodate colonic epithelium-bacterium coculture for multiple donors for up to 4 days, these experiments are performed with limited repeats due to COVID disruption. Further experiments are warranted to validate the broad applicability of the GuMI model.

### GuMI and *F. prausnitzii* alter the chemical profile

**Figure 2:**
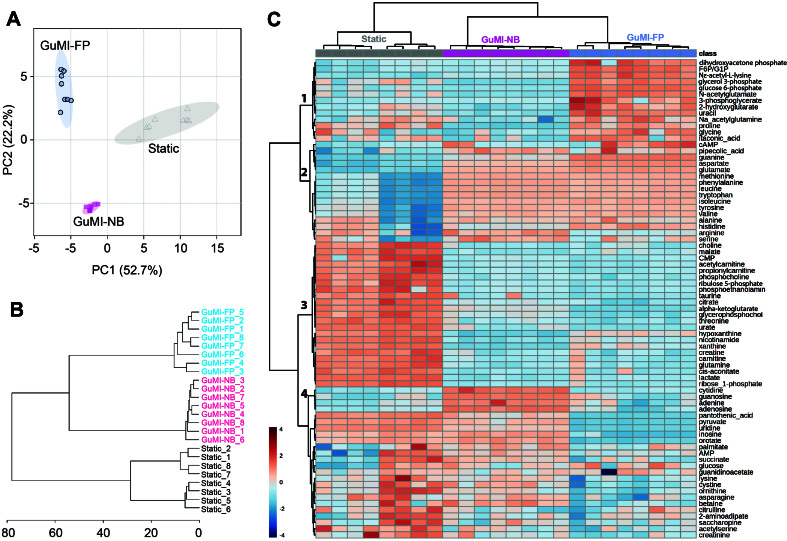
Metabolite profile in the apical compartment in static condition (Static) and GuMI culture with and without bacteria. (A) The principal component analysis plot indicates a clear separation of the analyzed metabolome among the three groups. In total, 76 detected compounds were included; (B) A distance plot of all the samples shows the inter-batch variation. In total, four batches of GuMI experiments were carried out. The samples are from four experiments as follows: experiment 1: Static_1 and 2, GuMI-NB_1, GuMI-FP_1 and 2; experiment 2: Static_3, 4, and 5, GuMI-NB_2 and 3, GuMI-FP_3 and 4; experiment 3: Static_6, GuMI-NB_4 and 5, GuMI-FP_5 and 6; experiment 4: Static_7 and 8, GuMI-NB_6, 7, and 8, GuMI-FP_7 and 8; (C) Heatmap of detected metabolites in Static, GuMI-NB, and GuMI-FP. Clusters 1, 2, 3, and 4 indicate the metabolites accumulated in specific conditions. GuMI-NB: GuMI without bacteria; GuMI-FP: GuMI with *F. prausnitzii*.

As demonstrated in our previous study, the characteristic metabolite butyrate was produced by *F. prausnitzii* in the apical compartment of GUMI^[15]^. As a proof of concept, we confirmed that the NF-κB pathway is suppressed by butyrate^[15]^. In addition to the NF-κB pathway, more than 4,000 genes were significantly changed by exposure to *F. prausnitzii*. This discrepancy leads to the hypothesis that *F. prausnitzii* alters other metabolites and influences the colonic epithelial transcriptome. To test this, we performed targeted metabolomic analysis (see Methods) to analyze the media collected from the apical compartment of the HC2978 monolayer under three conditions, i.e., Static culture, GuMI with no bacteria, and GuMI with *F. prausnitzii* (GuMI-FP, originated from the original name *F. prausnitzii*). Out of 169 chemicals analyzed, 76 metabolites were above the detection limit in at least one-third of the samples [Supplementary Tables 1 and 2]. Principal component analysis indicates a clear separation among the three conditions [Figure 2A]. Principal components 1 and 2 account for over 70% of the variance. We noted a variation within each group, as the replicates from the same experimental repeat tended to cluster [Figure 2B]. Statistical analysis identified metabolites that contribute to separating the three groups in the PCA plot. These chemicals clustered into several clusters attributed to Static, GuMI-NB, and GuMI-FP, respectively [Figure 2C]. For example, chemicals in cluster 1 were found to be at the highest level in GuMI-FP, suggesting that *F. prausnitzii* produces these metabolites. Additionally, metabolites in cluster 3 were at the highest level in Static culture, suggesting that these metabolites are produced by colonic epithelium and washed away by the flow in GuMI [Figure 2C]. Together, these results indicate that the GuMI coculture system, in combination with metabolomics, can effectively discern the influence of *F. prausnitzii*, epithelial cells, and flow on the metabolites in the apical compartment.

### Colonic epithelium consumes amino acids and secretes diverse metabolites related to the glycolysis pathway

**Figure 3:**
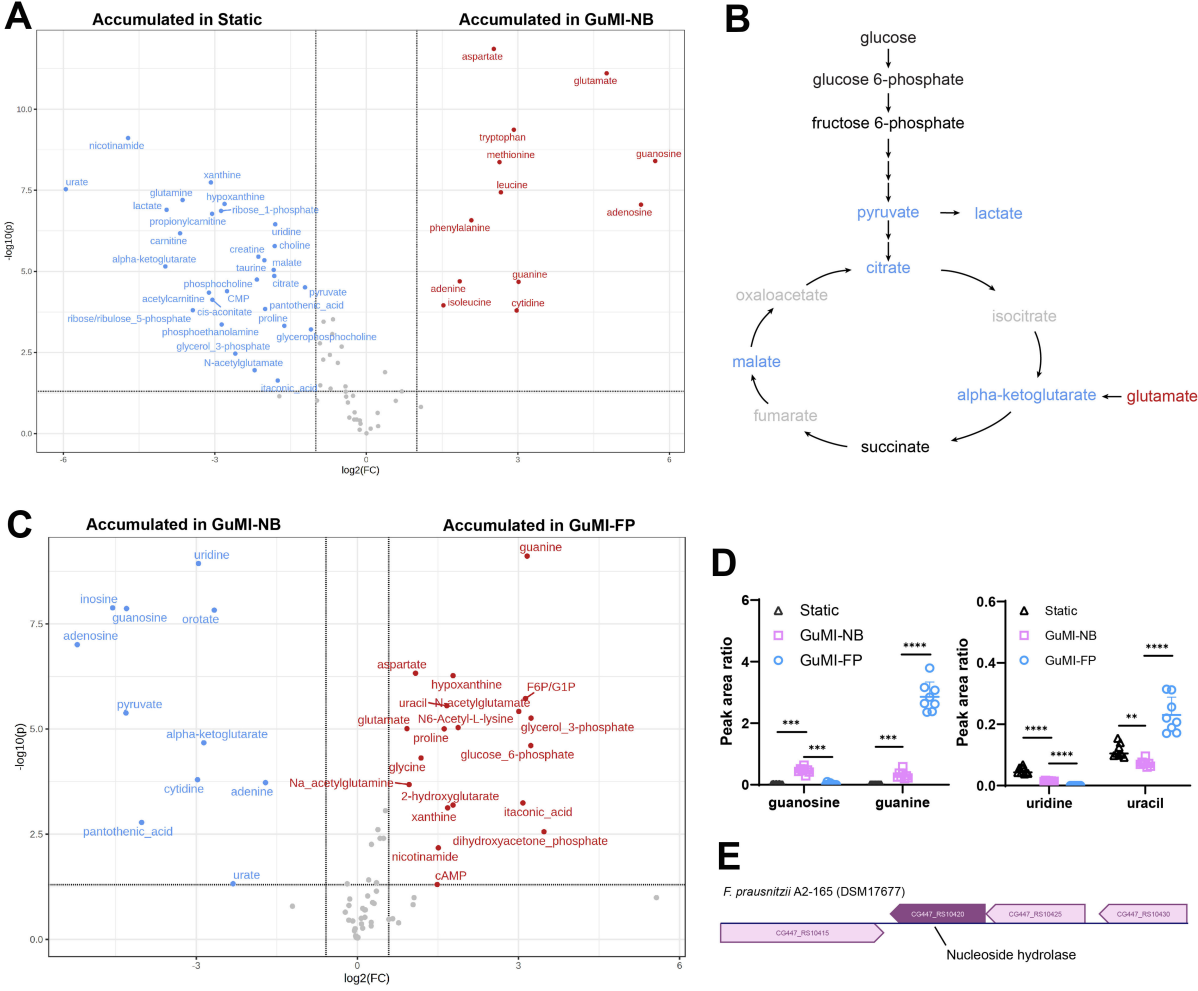
Metabolomics revealed specific metabolite changes attributed to the host, continuous flow, and *F. prausnitzii* A2-165. (A) The volcano plot of GuMI-NB *vs*. Static. *P* < 0.05, |fold change| > 2. Blue is significantly higher in static condition (Static) culture; red is significantly higher in GuMI-NB; (B) Metabolites mapped to glycolysis and Krebs cycle pathways. Metabolites highlighted in red, blue, and black are significantly higher, significantly lower, and not significantly changed in GuMI-NB *vs*. Static, respectively. Significance threshold: *P* < 0.05, |fold change| > 2. Metabolites in grey are not detected; (C) The volcano plot of metabolite changes in GuMI-FP *vs*. GuMI-NB. *P* < 0.05, |fold change| > 1.5. The rationale for lowering the threshold is that the continuous flow dilutes the produced metabolites. Therefore, a 50% change in the chemical concentration is considered significant; One-way ANOVA was performed for each compound in (B-D) to compare the differences between Static, GuMI-NB, and GuMI-FP. Note that the peak area ratio can not be used to compare the levels of different compounds; (E) Nucleoside hydrolase gene in the genome of *F. prausnitzii* A2-165. Gene ID: CG447_RS10420; accession ID of its predicted protein: C7H870 (UniProt). GuMI-NB: GuMI without bacteria; GuMI-FP: GuMI with *F. prausnitzii*.

Next, we sought to determine what metabolites were specifically consumed or secreted by colonic epithelial cells. Twelve metabolites were significantly higher in GuMI-NB than in Static (*P* < 0.05, fold change > 2, Figure 3A), and these metabolites belong to amino acids, nucleosides, and nucleobases. These results suggest that these compounds are primarily from the apical medium and consumed by colonic epithelial cells. Specifically, we found glutamate consumption is accompanied by the production of its metabolite alpha-ketoglutarate, which is accumulated in Static [Figure 3A]. Alpha-ketoglutarate can also be generated by glycolysis and Krebs cycle. Correspondingly, we found that pyruvate, lactate, citrate, alpha-ketoglutarate, and malate were significantly accumulated in Static culture [Figure 3B]. Other glycolysis and Krebs pathways metabolites were also detected but did not change significantly [Figure 3B]. Together, these results suggest that continuous flow in GuMI supplies amino acids and nucleosides for colonic epithelial cells and washes away many metabolites produced by colonic epithelial cell metabolism.

### *F. prausnitzii* metabolizes host- and nutrient-derived nucleosides, nucleobases, and amino acids in GuMI

In addition to lactate, *F. prausnitzii* can metabolize other compounds from the fresh medium or colonic epithelium. For example, guanosine did not accumulate in the Static culture but was reliably detected in GuMI-NB, indicating that it was mainly from the fresh medium and not from epithelial cells. However, the level of guanosine in GuMI-FP significantly (*P* < 0.0001) dropped by ~20 times to a low level [Figure 3C]. Correspondingly, guanine, the hydrolysis product of guanosine, increased by ~9 times in GuMI-FP compared to GuMI-NB (*P* < 0.0001, Figure 3C). The lower increase in guanine may hint that *F. prausnitzii* consumes it during the growth. On the other hand, uridine was at a higher level in Static culture than GuMI-NB, indicating that it is derived from epithelial cells. In GuMI-FP, the uridine level decreased to an undetectable level (zeros in all samples, Figure 3D, Supplementary Tables 1 and 2), while uracil, the hydrolysis product of uridine, increased by ~4 times compared to that in GuMI-NB. These results suggest that *F. prausnitzii* converted medium-derived guanosine and epithelium-derived uridine to their hydrolysis products, guanine and uracil. In agreement, only one nucleoside hydrolase-encoding gene was found in the genome of *F. prausnitzii* A2-165 [Figure 3E]. Other nucleosides and nucleobases were consumed entirely by *F. prausnitzii*, evidenced by the deficient levels of adenosine, adenine, and cytidine in GuMI-FP compared to GuMI-NB [Supplementary Tables 1 and 2]. In agreement, adenine, inosine, xanthosine, and 5’-methylthioadenosine were found to be consumed by *F. prausnitzii* in a Caco-2-*F. prausnitzii* coculture compared to Caco-2 monoculture^[24]^.

Beyond nucleoside and nucleobases, *F. prausnitzii* modified amino acids and amino acid-related metabolites in the apical compartment. The levels of proline, N-acetylglutamate, itaconic acid, 2-hydroxyglutarate, N-acetylglutamine, and N6-acetyllysine in GuMI-FP were significantly higher than that in GuMI-NB [Figure 3C]. Recently, it was shown that proline is essential to maintain gut homeostasis by activating lymphoid tissue inducer (LTi) cells^[25]^. Disruption of proline uptake in LTi cells impairs LTi cell activation and promotes DSS-induced colitis^[25]^. In addition, several intracellular intermediates of glucose and lipid metabolism, such as glucose-5-phosphate, F6P/G1P, and glycerol-3-phosphate [Figure 3C], increased significantly in GuMI-FP compared to those in other conditions, indicating that bacterial cell lysis occurred.

The modulation of nucleosides and amino acids was observed previously for *F. prausnitzii*. In a defined medium, *F. prausnitzii* consumed guanine, uracil, xanthine, orotic acid, histidine, leucine, tryptophan, phenylalanine, and nicotinamide while producing L-glutamine and L-threonine.^14^ In the GuMI experiment, histidine, leucine, tryptophan, phenylalanine, and nicotinamide were also detected in GuMI-NB but not significantly changed by *F. prausnitzii* in GuMI-FP. This disagreement might be due to different experimental settings, i.e., a static culture of *F. prausnitzii* alone in a defined medium *vs*. a fluidic coculture of *F. prausnitzii* with epithelial cells in an undefined YCFA medium. Nevertheless, most of the metabolites detected *in vitro* in the GuMI platform were also observed in the human intestine *in vivo*. For instance, guanosine, guanine, uracil, adenosine, cytidine, amino acids, and amino acid metabolites [Supplementary Tables 1 and 2] were detected in human fecal samples^[26,27]^. Together, these results indicate *F. prausnitzii* may play an essential role in modulating chemicals in the human intestinal lumen. Whether these modifications by *F. prausnitzii* translate into clinical settings warrants more well-controlled clinical trials^[28]^.

### Transcriptional changes of transcription factors in colonic epithelium linked to their ligands metabolized by *F. prausnitzii*

**Figure 4:**
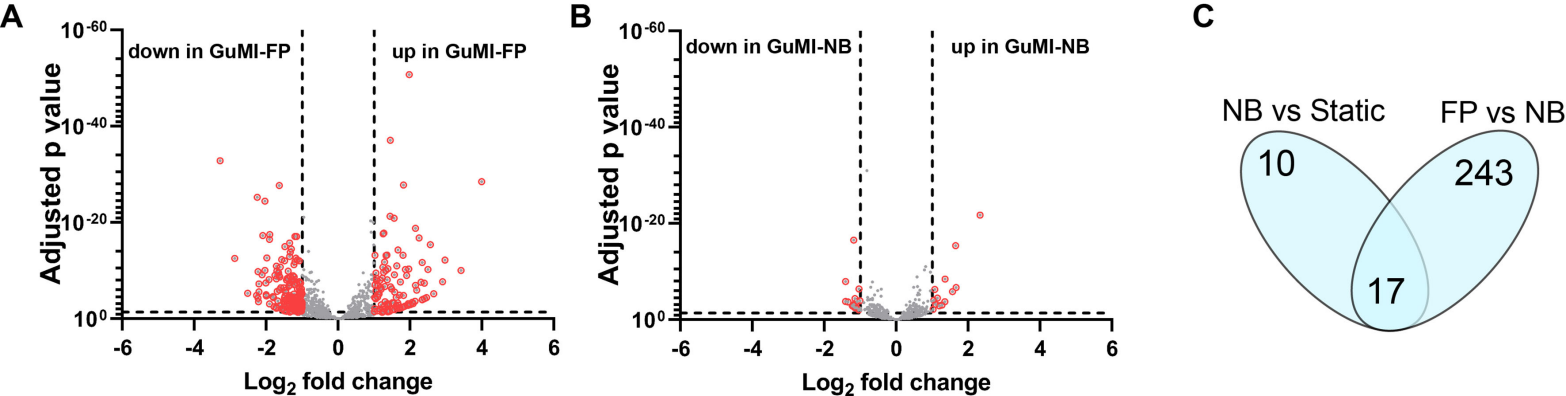
*F. prausnitzii* has a higher impact than the microenvironment on the expression of transcription factor genes in the colonic epithelial cells. (A)Volcano plot on the gene expression of transcription factors in GuMI-FP *vs*. GuMI-NB. The 260 significantly changed genes (*P* < 0.05, log2(|FC|) > 1) are highlighted in red; and (B) volcano plot on the gene expression of transcription factors in GuMI-NB *vs*. GuMI-FP. The 27 significantly changed genes (*P* < 0.05, log2(|FC|) > 1) are highlighted in red; (C) Venn plot on the significantly changed transcription factor indicates the unique effects of *F. prausnitzii*. Seventeen transcription factors are changed by microenvironment change (NB *vs*. Static) and *F. prausnitzii* (FP *vs*. NB). GuMI-FP: GuMI with *F. prausnitzii*; GuMI-NB: GuMI without bacteria.

Transcription factors (TFs) in the host cells are master regulators of host-microbe, host-virus, and host-pathogen interactions because transcription factors profoundly influence gene transcription in the downstream pathways. As *F. prausnitzii* changed the levels of many metabolites beyond butyrate, we hypothesize that more transcription factors are modulated by *F. prausnitzii* and the microenvironment in GuMI (e.g., oxygen gradient and flow). Hence, identifying the *F. prausnitzii*-transcription factor gene interaction may offer new insights into the beneficial effects of *F. prausnitzii*. To test this, we re-analyze the transcriptomic data in our previous study^[15]^. Among 1,665 known human transcription factors obtained from Human Transcription Factor Database (http://bioinfo.life.hust.edu.cn/HumanTFDB), 1,351 were detected, and 260 were significantly changed (|log2 fold change| ≥ 1, adjusted *P* ≤ 0.05) in the epithelial cells in GuMI-FP *vs*. GuMI-NB [Figure 4A, Supplementary Tables 3 and 4]. In contrast, only 27 TF genes were changed considerably in GuMI-NB *vs*. Static culture, despite a similar number of TF genes (1,306) being detected [Figure 4B, Supplementary Tables 3 and 4]. Among all changed TF genes, only 10 TFs are specific in GuMI-NB. These results suggest that despite the apical compartment being switched from Static and oxygenated to a fluidic and anerobic environment, it induces relatively mild effects on the transcriptome of colonic epithelial cells. In contrast, *F. prausnitzii* specifically altered the expression of 243 TFs in the human colonic epithelial cells in GuMI [Figure 4C]. Many of these TFs belong to the families involved in cell metabolism (bHLH) and cell differentiation (Homeobox). For instance, the disheveled binding antagonist of beta-catenin 3 (*DACT3*) and *FOSL1* are genes linked to canonical and non-canonical Wnt signaling pathways essential for cell differentiation. Here, we found that *DACT3* and *FOSL1*, transcription factor genes *JUNB* and *JUND* in colonic epithelial cells were significantly increased by *F. prausnitzii* in GuMI-FP.

Interestingly, we also found that interferon transcription factors *IRF1*, *IRF6*, and *IRF7* were significantly increased in GuMI-FP. IRF7 is a master transcription factor of Type I interferon-dependent immune responses^[29]^, making it a potential target for infection control^[30]^. Notably, early growth response 1 (*EGR1*) transcription is significantly reduced by *F. prausnitzii* in GuMI [Supplementary Tables 3 and 4].

## DISCUSSION

**Figure 5:**
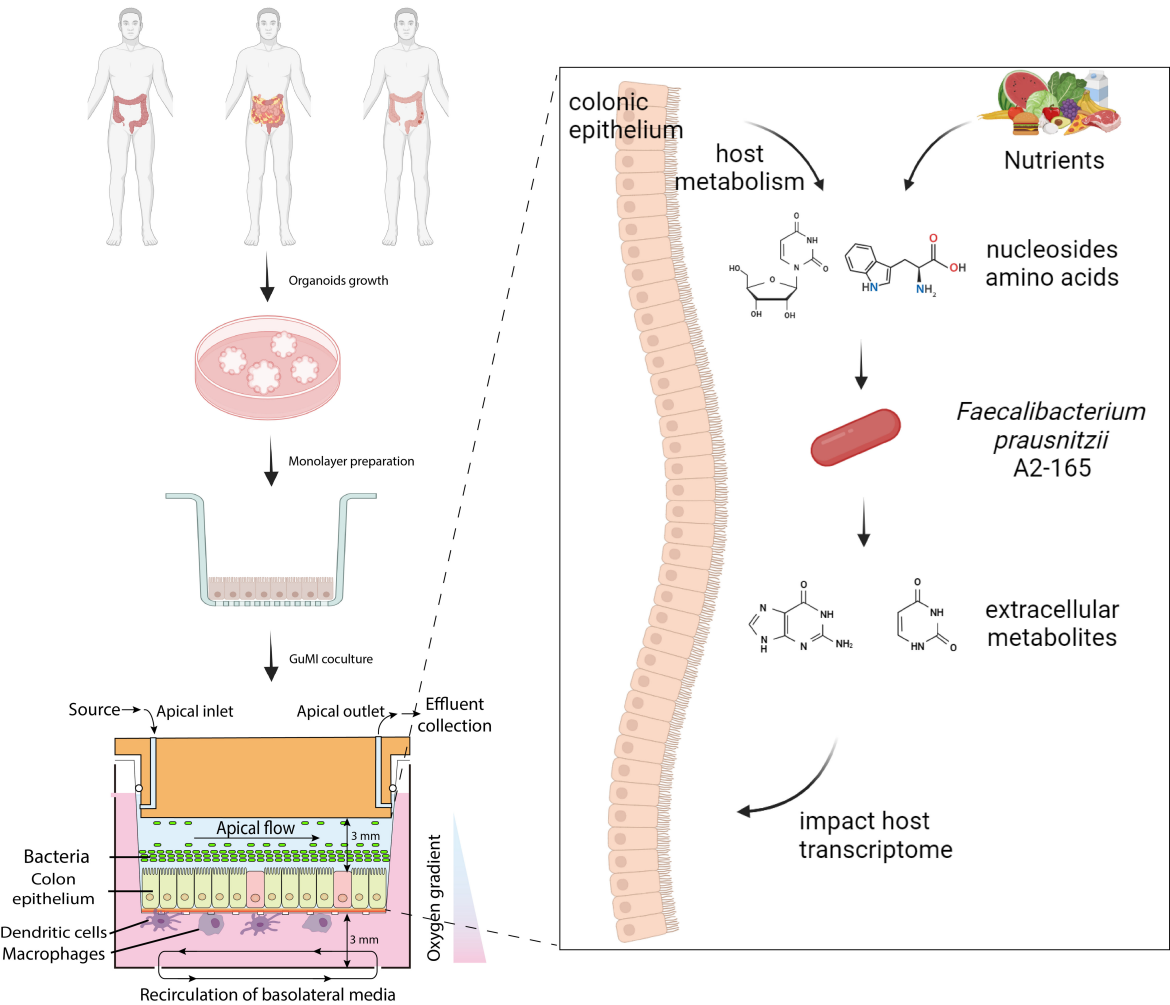
Overview of using GuMI microphysiological system and metabolomics to identify new metabolic activities of *F. prausnitzii* in modulating chemical profile in the intestinal lumen and inducing a transcriptional change in colonic epithelial cells.

We demonstrated that the GuMI microphysiological system is feasible for multiple donors from healthy individuals and patients of IBD. Using metabolomics, we found that *F. prausnitzii* dramatically modulates the chemicals derived from the host and medium. Coculture of *F. prausnitzii* with colonic epithelium induces specific transcriptional changes of transcription factor genes in the colonic epithelium [Figure 5]. These results suggest that metabolomics, in combination with the GuMI microphysiological system, enables us to discover novel metabolic activities of *F. prausnitzii* that may contribute to the host-microbiome crosstalk in the human intestine.

Similar to our observations, Lenoir *et al*. found that *F. prausnitzii* supernatant induces the expression of *DACT3 in vitro* in HT-29 cells and *in vivo* in mice, and identified butyrate as the effector metabolite^[11]^. Indeed, butyrate was produced and maintained at mM level in GuMI-FP^[15]^. Contradictory to these findings, Lukovac *et al*. found that *F. prausnitzii* supernatant has a minimal effect on the gene expression of mouse ileal organoids despite a high level of butyrate (8.03 mM) in the supernatant^[31]^. Multiple variants could explain this discrepancy in the experiments with GuMI *vs*. organoids, including cell sources (human colon *vs*. mouse ileum), exposure time (48 h *vs*. 3 h), exposure routes (continuous exposure on the apical side *vs*. static exposure on the basolateral side), and microenvironment (anoxic-oxic gradient *vs*. oxic). Future research needs to clarify this discrepancy by taking these factors into account.

Gut microbiota are known to mediate resistance to infections through multiple mechanisms^[32]^. Here, we observed that *F. prausnitzii* significantly changed the expression of transcription factors in colonic epithelial cells, including *IRF*s and *EGR1* related to viral and bacterial infection. Pathogenic bacteria such as *Pseudomonas aeruginosa*, *Staphylococcus aureus*, *Salmonella* strains, and *E. coli* can upregulate *EGR1* in different host epithelial cells^[33]^, independent of bacterial adherence to epithelial cells. The downregulation of *EGR1* and increase of *IRF*s by *F. prausnitzii* might serve as new mechanisms contributing to the pathogen resistance of the gut in a homeostatic scenario.

While it is not fully understood how *F. prausnitzii* precisely modifies the expression of TF genes in colonic epithelium, several mechanisms may contribute to the observed effects. For instance, short-chain fatty acids and amino acid metabolites can directly modify histones such as methylation, acetylation, and serotonylation, influencing chromatin accessibility and gene transcription^[34]^. In addition, gut microbial metabolites can directly bind G-protein coupled receptors^[3,35]^ and trigger downstream signaling pathways such as the activation of transcription factor NF-κB. It is worth noting that only 169 metabolites were included in the target metabolomic analysis, which is a limitation of this study. Hence, including more metabolites and other bacterial components such as proteins, lipids, and cell wall components in the analysis will provide a more comprehensive view of how *F. prausnitzii* impacts TF transcription.

## DECLARATIONS

### Availability of data and materials

The data supporting this study’s findings are available in the Supplementary Materials or from the corresponding authors upon request.

### Copyright

© The Author(s) 2024.
