# Supplementary material for: Faecalibacterium prausnitzii A2-165 metabolizes host- and media-derived chemicals and induces transcriptional changes in colonic epithelium in GuMI human gut microphysiological system
Source: Microbiome Res Rep. 2024 May 22;3(3):30. doi: 10.20517/mrr.2024.14 (PMC11480719; doi:10.20517/mrr.2024.14)
Supplement: Supplementary file 1 [file mrr-3-3-30-SupplementaryMaterials.pdf]

Huang *et al. Microbiome Res Rep* 2024;3:30
DOI: 10.20517/mrr.2024.14

Microbiome Research Reports

Supplementary Material

Faecalibacterium prausnitzii A2-165 metabolizes host- and media-derived chemicals and induces transcriptional changes in colonic epithelium in GuMI human gut microphysiological system

Yu-Ja Huang[1], Caroline A. Lewis[2,3], Charles Wright[1], Kirsten Schneider[1], John Kemmitt[1], David L. Trumper[4], David T. Breault[5], Omer Yilmaz[1], Linda G. Griffith[1,4,6], Jianbo Zhang[1,7,8]

[1]Department of Biological Engineering, Massachusetts Institute of Technology, Cambridge, MA 02139, USA.
[2]Whitehead Institute for Biomedical Research, Cambridge, MA 02142, USA.
[3]current address: UMass Chan Medical School, Program in Molecular Medicine, Worcester, MA 01605, USA.
[4]Department of Mechanical Engineering, Massachusetts Institute of Technology, Cambridge, MA 02139, USA.
[5]Department of Pediatrics, Harvard Medical School, Boston, MA 02115, USA.
[6]Center for Gynepathology Research, Massachusetts Institute of Technology, Cambridge, MA 02139, USA.
[7]Swammerdam Institute for Life Sciences, University of Amsterdam, Amsterdam 1098 XH, the Netherlands.
[8]Tytgat Institute for Liver and Intestinal Research, Amsterdam Gastroenterology, Endocrinology and Metabolism, Amsterdam UMC, Location Academic Medical Center, Amsterdam 1105 BK, the Netherlands.

Correspondence to: Dr. Jianbo Zhang, Swammerdam Institute for Life Sciences, University of Amsterdam, Science Park 904, Amsterdam 1098 XH, the Netherlands. E-mail: j.zhang6@uva.nl **ORCID:** Jianbo Zhang (0000-0003-3526-4586)

© The Author(s) 2024. Open Access This article is licensed under a Creative Commons Attribution 4.0 International License (https://creativecommons.org/licenses/by/4.0/), which permits unrestricted use, sharing, adaptation, distribution and reproduction in any medium or format, for any purpose, even commercially, as long as you give appropriate credit to the original author(s) and the source, provide a link to the Creative Commons license, and indicate if changes were made.

OAE Publishing Inc.

www.mrrjournal.net

MAIN TEXT

Organoids, monolayers, and GuMI experiments

To establish organoid cultures, endoscopic tissue biopsies were collected from the colon of deidentified individuals at either Massachusetts General Hospital or Boston Children's Hospital upon the donors' informed consent. Methods were carried out following the Institutional Review Board of Boston Children's Hospital (protocol number IRB-P00000529) and the Koch Institute Institutional Review Board Committee, as well as the Massachusetts Institute of Technology Committee on the use of humans as experimental subjects. In brief, organoids in Matrigel (growth factor reduced, phenol red-free; Corning, 356231) droplets were grown in 24-well tissue culture-treated plates (Olympus Plastics, 25-107) and passaged every seven days at a 1:3 split ratio. A medium change was performed on day four or every another day using organoid growth medium after passaging (subject to optimization for each donor). To prepare the monolayer, organoids were collected on day 7 and pelleted by centrifugation (1000 *g* × 5 min, 4°C), followed by digestion using Cell Recovery Solution (Corning, 354253; 1 mL per 100 µL Matrigel). The resulting organoid suspension was then incubated on ice for 45-60 min, pelleted, and then digested at 37 °C for 5 min using 1 mL Trypsin/EDTA (2.5 mg/mL Trypsin [Sigma, T4549] and 0.45 mM EDTA [Ambion, AM9260G] in PBS without calcium and magnesium [$PBS^{-/-}$, Gibco, 10010-023]). The digested organoids were manually dissociated into single cells using a 1000-µL pipette with a bent tip. The resulting cell suspension was then pelleted (300 *g* × 5 min, 4 °C) after neutralizing Trypsin was neutralized with 10% FBS in the base medium. The cell pellet was resuspended in the seeding medium and seeded in collagen I-coated (Gibco, A10483-01, 50 µg/mL in PBS) 12-well Transwells (Corning 3460, 0.4 µm pore polyester membrane). The seeding cell density was 300,000 cells per well (surface area: 1.12 cm^2). Around 72 h after seeding, the monolayers were differentiated by switching to the antibiotic-free base medium on the apical side and the differentiation medium on the basolateral side. After switching to a differentiation medium, the monolayers were cultured for four days (a total of 7 days), with medium change on day 5. The monolayers were used for experiments on day seven after seeding (day four after differentiation).

For GuMI experiments, differentiated monolayers were integrated in GuMI device for

24 h before adding bacteria. The overnight grown bacterial cultures were diluted 1000 times with a pre-reduced YCFA medium. After that, 0.8-1 mL of the diluted bacterial cells were slowly injected into GuMI[1,2]. After bacterial injection, the coculture was settled for 1 h and resumed at the designed flow rate and period. At the end of the experiments, samples were collected, and TEER measurements were performed based on the previous report[1].

Isolation and differentiation of monocytes

When macrophages and dendritic cells were included, monocytes were isolated from PBMCs and differentiated into macrophages and dendritic cells based on our previous study[2]. Monocytes were isolated from PBMC. PBMC were isolated from fresh whole blood with CPDA-1 anticoagulant (Research Blood Components LLC) using the SepMate PBMC Isolation Kit (StemCell, 85450) following the manufacturer protocol. After isolation, the PBMC were suspended in an immune cell freezing medium (RMPI with 10% dimethyl sulfoxide [DMSO] and 10% heat-inactivated FBS) and frozen at -196 °C.

Monocytes were isolated and differentiated into dendritic cells and macrophages on the 7th day before device assembly for 7 days. First, the PBMC were thawed in a 37 °C water bath for ~1 min before diluting 1:10 in $PBS^{-/-}$ containing 2% heat-inactivated FBS (PBS-HIFBS). After that, cells were pelleted (300 *g* × 5 min, 4 °C) and then resuspended in 1 mL of PBS-HIFBS, followed by a transfer into a 5-mL round-bottom polystyrene tube (StemCell, 100-0088). An additional 1.5 mL PBS-HIFBS was used to recover the residual cells and transferred into the same polystyrene tube. The isolation of monocytes was performed using the EasySep™ Human Monocyte Enrichment Kit without CD16 depletion (StemCell, 19058) and EasySep™ Magnet (StemCell, 18000). The resulting monocytes were split into two aliquots and pelleted (300 *g* × 5 min, 4 °C). The cell pellets were resuspended in the macrophage or dendritic cell differentiation media and cultured in 24-well tissue-treated plates. Both dendritic cells and macrophages were plated at a density of 1×10^6 cells per well and in a volume of 500 µL per well. Four days after isolation and plating, 500 µL of Macrophage Differentiation Medium and Dendritic Cell Differentiation Medium [Supplementary Table 1] were added to each macrophage and dendritic cell well, respectively.

In the experiments with dendritic cells and macrophages, cells were harvested and seeded onto the basolateral side of the transwell membrane. To harvest the cells, cells were resuspended in their own media and collected into a conical tube (one tube per cell type). The residual cells were detached by adding 250 µL TrypLE Express (Gibco, 1260413) to each well and incubated at 37 °C for ~15 min, or until cells were detached from the plate. The TrypLE was then neutralized with 750 µL RPMI-HIFBS. The resulting cell suspensions were collected into the corresponding conical tubes. After that, the cells were pelleted (300 *g* × 5 min, 4 °C), resuspended in 1 mL of RPMI-HIFBS, and counted using trypan blue and countess. Dendritic cells and macrophages were then combined to achieve a density of 1.67×10^5 cells per mL for each cell type. Before adding dendritic cells and macrophages, the media of the transwells was removed from both apical and basolateral sides, and each side was rinsed once with an antibiotic-free base medium. The transwells were then inverted and placed in a petri dish before adding 150 µL of the dendritic cell and macrophage cell suspension to each well to achieve a density of 0.25×10^5 cells per transwell for each cell type. The transwells were then incubated at 37 °C for 2 h to allow the attachment of dendritic cells and macrophages before proceeding with the further experimental setup.

Composition of media used in this study

Base Medium: Advanced DMEM/F12, 2mM Glutamax, 10mM HEPES, 1x Penicillin/Streptomycin.

Antibiotic-free Base Medium: Advanced DMEM/F12, 2mM Glutamax, 10mM HEPES

Organoid Growth Medium: 65% L-WRN conditioned medium, 32% base medium, 1x B-27, 1x N-2, 10 mM Nicotinamide, 500 µM N-acetyl L-cysteine, 10 µM Y-27632 dihydrochloride, 10 µM SB202190, 500 nM A83-01, 50 ng/mL murine EGF, 10 nM human [Leu^{15}]-Gastrin I, 5 nM prostaglandin E2.

Washing Medium: DMEM/Nutrient Mixture F-12 Ham, 10% heat-inactivated FBS, 2mM glutamax, 1x Penicillin/Streptomycin.

Colon Seeding Medium: 65% L-WRN conditioned medium, 32% base medium, 1x

B-27, 1x N-2, 500 µM N-acetyl L-cysteine, 10 µM SB202190, 500 nM A83-01, 2.5 µM thiazovivin, 50 ng/mL murine EGF, 10 nM human [Leu15]-Gastrin I, 5 nM prostaglandin E2.

Colon Differentiation Medium: 20% R-spondin1 conditioned medium, 80% antibiotic-free base medium, 1x B27, 1x N2, 500 µM N-acetyl-L-cysteine, 500 nM A83-01.

Dendritic Cell Differentiation Medium: 90% RPMI 1640, 10% heat-inactivated FBS, 100 ng/mL GM-CSF/CSF2, 70 ng/mL recombinant human IL-4, 10 nM retinoic acid.

Macrophage Differentiation Medium: 90% RPMI 1640, 10% heat-inactivated FBS, 100 ng/mL M-CSF/CSF1

REFERENCES

1. Zhang J, Huang YJ, Yoon JY, *et al.* Primary human colonic mucosal barrier crosstalk with super oxygen-sensitive *Faecalibacterium prausnitzii* in continuous culture. *Med* 2021;2:74-98. [PMID:33511375 DOI:10.1016/j.medj.2020.07.001 PMCID:PMC7839961]
2. Zhang J, Huang YJ, Trapecar M, *et al.* An immune-competent human gut microphysiological system enables inflammation-modulation by *Faecalibacterium prausnitzii*. *npj Biofilms Microbiomes* 2024;10:31. [DOI:10.1038/s41522-024-00501-z]
